# Effect of Gut Microbiota on the Pharmacokinetics of Nifedipine in Spontaneously Hypertensive Rats

**DOI:** 10.3390/pharmaceutics15082085

**Published:** 2023-08-03

**Authors:** Rong Zhou, Haijun Yang, Peng Zhu, Yujie Liu, Yanjuan Zhang, Wei Zhang, Honghao Zhou, Xiong Li, Qing Li

**Affiliations:** 1 Department of Clinical Pharmacology, Xiangya Hospital, Central South University, Changsha 410008, China; 2 Institute of Clinical Pharmacology, Hunan Key Laboratory of Pharmacogenetics, Central South University, Changsha 410078, China; 3 Engineering Research Center of Applied Technology of Pharmacogenomics, Ministry of Education, Changsha 410078, China; 4 National Clinical Research Center for Geriatric Disorders, Changsha 410008, China; 5 Key Specialty of Clinical Pharmacy, The First Affiliated Hospital of Guangdong Pharmaceutical University, Guangzhou 510699, China

**Keywords:** gut microbiota, nifedipine, pharmacokinetics, SHR, CYP3A1

## Abstract

The pharmacokinetic variability of nifedipine widely observed in the clinic cannot be fully explained by pharmacogenomics. As a new factor affecting drug metabolism, how the gut microbiota affects the pharmacokinetics of nifedipine needs to be explored. Spontaneously hypertensive rats (SHRs) have been commonly used in hypertension-related research and served as the experimental groups; Wistar rats were used as control groups. In this study, the bioavailability of nifedipine decreased by 18.62% (*p* < 0.05) in the SHRs compared with the Wistar rats. Changes in microbiota were associated with the difference in pharmacokinetics. The relative abundance of *Bacteroides dorei* was negatively correlated with AUC_0–t_ (*r* = −0.881, *p* = 0.004) and *C*_max_ (*r* = −0.714, *p* = 0.047). Analysis of serum bile acid (BA) profiles indicated that glycoursodeoxycholic acid (GUDCA) and glycochenodeoxycholic acid (GCDCA) were significantly increased in the SHRs. Compared with the Wistar rats, the expressions of CYP3A1 and PXR were upregulated and the enzyme activity of CYP3A1 increased in the SHRs. Spearman’s rank correlation revealed that *Bacteroides stercoris* was negatively correlated with GUDCA (*r* = −0.7126, *p* = 0.0264) and GCDCA (*r* = −0.6878, *p* = 0.0339). Moreover, GUDCA was negatively correlated with *C*_max_ (*r* = −0.556, *p* = 0.025). In primary rat hepatocytes, GUDCA could induce the expressions of PXR target genes *CYP3A1* and *Mdr1a*. Furthermore, antibiotic treatments in SHRs verified the impact of microbiota on the pharmacokinetics of nifedipine. Generally, gut microbiota affects the pharmacokinetics of nifedipine through microbial biotransformation or by regulating the enzyme activity of CYP3A1.

## 1. Introduction

Nifedipine is the earliest dihydropyridine calcium channel blocker used in antihypertensive therapy, ref. [1] which is almost completely absorbed in the gastrointestinal tract with the characteristics of low solubility and high intestinal permeability [2] and predominantly metabolized by CYP3A in the liver [3]. A population study of nifedipine indicated that great interindividual variability existed in the oral clearance of nifedipine [4]. In a double-blind trial, 49 patients received nifedipine 20 mg twice daily; after 2 or 4 weeks, 56% of patients required a higher dose (40 mg twice daily), indicating the interindividual heterogeneity in drug response [5].

Pharmacokinetics are affected by the process of drug disposition in the body, including absorption, distribution, metabolism, and excretion. The gut microbiota, as an important factor affecting pharmacokinetics, has aroused widespread attention [6]. The human gut is home to about 10^13^–10^14^ bacteria containing three million microbial genes, more than 100 times the number of human genes [7]. Gut microbiota as a metabolic “organ” can participate in drug metabolism through direct or indirect interactions to alter its bioavailability, activity, or toxicity and influence the individual’s response to drugs [8]. Direct interactions include the conversion of part or all of the drugs to metabolites by microbiome-derived enzymes [9]. *Eggerthella lenta* can reduce digoxin in vitro, and pharmacokinetic studies in vivo revealed that diet could reduce the microbial metabolism of digoxin [10]. Moreover, *Bacteroides thetaiotaomicron* can metabolize diltiazem into diacetyl diltiazem [11]. Indirect interactions include the effects of microbial-derived metabolites on host metabolism [12]. Yang et al. [13] found the antibiotics-induced depletion of rat microbiota influenced the expression of host drug-processing genes. Zhou et al. [14] reported that intestinal flora affected the metabolism of CSA by altering the expression of CYP3A1, UGT1A1, and P-GP. Secondary bile acids, enteric microbiome metabolites, were positively related to the concentration of simvastatin [15].

Bile acids are an important class of microbially produced metabolites [16]. Gut microbiota mediates the production of bile acids via bile salt hydrolases (BSH), hydroxysteroid dehydrogenases (HSDH), 7-α-HSDH, 7-α-dehydroxylase, and taurine metabolism. Bile acids, as the endogenous ligands of some nuclear receptors, including PXR, farnesoid X receptor (FXR), vitamin D3 receptor (VDR), and G-protein-coupled bile acid receptor (TGR5), can regulate related signal pathways and not only affect their synthesis and secretion but also alter host metabolism [17,18]. Secondary bile acids LCA and 3-keto-LCA have been reported to be agonists of PXR, and the activation of the PXR–CYP3A4 axis in the liver can promote drug metabolism [19].

Hypertension is the most prevalent chronic disease and is also a preventable risk factor for stroke, heart failure, and other serious cardiovascular and cerebrovascular diseases [20]. Blood pressure control rates need to be improved effectively [21,22]. An increasing body of evidence supports the correlation between gut microbiota and hypertension in patients [23,24,25,26] and animal models [27,28,29]. SHRs (spontaneously hypertensive rats) are an ideal model for the study of human primary hypertension, which was established by selective inbreeding and acquired the trait of spontaneous hypertension and polygenic [30].

Considering that the pharmacokinetic variability of nifedipine has not been fully explained, while microbial metabolism as a potential mechanism needs to be further explored [31], the objective of this study is to explore how gut microbiota directly or indirectly affects the pharmacokinetics of nifedipine.

## 2. Materials and Methods

### 2.1. Materials

Materials used in this study were nifedipine (N7634, 100%; Sigma, St. Louis, MO, USA); nitrendipine (B27254, >98%; YuanYe, Shanghai, China); and dehydronifedipine (ZTL-N-074, >98%; SHANGHAI ZZBIO Co., Ltd., Shanghai, China). The sources of antibodies were as follows: CYP3A1 (sc-53246, Santa Cruz Biotechnology, Dallas, TX, USA); PXR (ab192579, Abcam, Cambridge, MA, USA); and GAPDH monoclonal antibody (60004-1-Ig, Proteintech, Wuhan, China).

### 2.2. Pharmacokinetic Study in Wistar Rats and SHRs

#### 2.2.1. Animals

Male 8-week-old SHRs and Wistar rats weighing about 200× *g* (certificate: SCXK (JING) 2016-0006) were purchased from the Beijing Vital River Laboratory Animal Technology Co., Ltd. (Beijing, China). All animals were kept in a 12 h light and dark cycle, temperature 21–22 °C, humidity 50–70%, and freely allowed laboratory animal maintenance feed (#MD17121, Medicience Ltd., Yangzhou, China) and water. Blood pressure was measured after 2 weeks of adaptive feeding, while systolic blood pressure (SBP) > 150 mmHg of SHRs was available for the study. The rats were divided into two groups: Wistar rats (control group) and SHRs (experimental group), *n* = 14 for each group. At 12 weeks, stool and serum samples were collected before pharmacokinetic experiments and stored at −80 °C until analysis. All animal experiments were approved by the experimental animal management committee and ethics committee of Central South University and complied with relevant rules and regulations (permit number 2020sydw1052).

#### 2.2.2. Pharmacokinetic Experiments

The two groups of rats were treated with the same method: after fasting overnight, 20 mg/kg of nifedipine (0.5% CMC–Na solution as solvent) was given to the rats by intragastric gavage. Blood samples were taken from the rats at 0, 0.167, 0.33, 0.5, 0.75, 1, 2.33, 4, 6, 8, and 24 h [32]. After the pharmacokinetic experiments, the rats were sacrificed by intraperitoneal injection of pentobarbital. The blood samples were centrifuged at 1681× *g* at 4 °C for 10 min to isolate plasma to determine the concentration of nifedipine.

#### 2.2.3. UPLC-MS/MS

Compounds were extracted from the plasma samples using the albumen precipitation method. UPLC-MS/MS detection was performed on ACQUITY UPLC M-Class (Waters Corp., Milford, MA, USA) and an API 4000 triple quadrupole tandem mass spectrometer (AB Sciex, Framingham, MA, USA). The chromatographic column was a HyPURITY C18 column (150 mm × 2.1 mm, SN: 10166977, Lot: 12782, Thermo Scientific, Waltham, MA, USA); oven temperature was maintained at 40 °C. The mobile phase consisted of acetonitrile (solvent A) and 0.1% formic acid water (solvent B). A gradient program was used for the HPLC separation: 0–1.5 min, 50% A and 50% B, 0.30 mL/min; 2.0–4.0 min, 70% A and 30% B, 0.40 mL/min; and 4.5–6.5 min, 50% A and 50% B, 0.30 mL/min. Quantitation was carried out using multiple reaction monitoring. The precursor–product ion pairs monitored were *m*/*z* 347.2 → 315.2 for nifedipine (DP 67 V, CE 12 eV), *m*/*z* 361.3 → 315.2 for nitrendipine (DP 75 V, CE 15 eV), and *m*/*z* 345.2→284.7 for dehydronifedipine (DP 89 V, CE 36 eV) (Supplementary Figure S1). Data were acquired from the Analyst 1.4.2 software (AB Sciex, Framingham, MA, USA).

### 2.3. Gut Microbiome Analysis

The total DNA was isolated from stool samples, and the universal primers of the V3–V4 region (341F: CCTACGGGRBGCASCAG; 806R: GGACTACNNGGGTATCTAAT) were adopted [33] for PCR amplification. PCR products were purified, quantified, and homogenized to form a sequencing library. The built library was subjected to library quality inspection, and the qualified library was sequenced with Illumina NovaSeq 6000.

The sequencing data were processed by Illumina bcl2fastq software (v2.20) for base calling, then spliced by fastq join (version: 1.3.1) and pear (v0.9.11) to obtain sequenced reads. Sequenced reads were cut and filtered using Cutadapt (version 1.18) to obtain the optimized sequence, namely clean tags. USEARCH software (version 11.0.667) was used to cluster operational taxonomic units (OTUs) according to 97% similarity sequences. The OTUs were annotated by the Silva database based on the representative sequences, and KronaTools was used to visually display the results of species annotation. The abundance of different taxonomy levels was generated by QIIME. α-diversity was processed using Mothur software (version 1.45.0). The R vegan package and gunifrac package were applied for β-diversity analysis. The R package phyloseq was used for PCoA analysis based on the Bray–Curtis distance. Line discriminant analysis effect size (LEfSe) was used to evaluate the effect size of differential features (i.e., LDA score).

### 2.4. Analysis of Bile Acids

Quantitation of bile acids was performed as previously described with minor modifications [34,35]. Serum samples were prepared with protein precipitation. Isotopic-labeled bile acids (C/D/N Isotopes, Quebec, Canada; Steraloids, Newport, RI, USA) were used as internal standards to quantify the content of bile acids. UPLC-MS/MS, ACQUITY UPLC Xevo TQ-S (Waters Corp., Milford, MA, USA), was applied to measure the content of bile acids. The chromatographic column was an ACQUITY UPLC Cortecs C18 1.6 µm VanGuard pre-column (2.1 mm × 5 mm) and ACQUITY UPLC Cortecs C18 1.6 µm analytical column (2.1 mm × 100 mm). The column temperature was 30 °C and the flow rate was 0.40 mL/min. The mobile phase was phase A (10 mM ammonium acetate with 0.25% acetic acid) and phase B (acetonitrile:methanol:isopropanol [8:1:1]) with gradient elution. The raw data were analyzed and processed using Masslynx software (v4.1, Waters, Milford, MA, USA).

### 2.5. RNA Extraction and Quantitative Real-Time PCR

Total RNA was extracted from the liver using RNAiso Plus (9109, TaKaRa, Kyoto, Japan) according to the manufacturer’s protocol. RNA concentration and purity were measured by UV–visible spectrophotometry, and when OD260/OD280 was between 1.8 and 2.0, it was qualified for RT-PCR. The 2 μg of total RNA was reversely transcribed into cDNA with a PrimeScript RT reagent kit (RR047A, TaKaRa, Kyoto, Japan). In addition, 2× SYBR Green qPCR Master Mix (B21203, Bimake, Houston, TX, USA) was used for RT-PCR. The relative expression of the target gene was evaluated by comparison with the PCR cycle threshold (Ct). *GAPDH*, as an internal reference, was used to normalize the expression of target genes. The primer sequences were acquired from the literature and are shown in Supplementary Table S1.

### 2.6. Western Blot

Western blot was performed as previously described [36]. Total protein was extracted from liver tissue with RIPA lysis buffer (P0013B, Beyotime, Shanghai, China) containing 1mM PMSF (ST507, Beyotime, Shanghai, China). The protein concentration was detected with a BCA protein assay kit (P0012S, Beyotime, Shanghai, China). The 20 μg protein samples were separated using electrophoresis in 10% sodium dodecyl sulfate–polyacrylamide gel (SDS-PAGE) and transferred to a polyvinylidene fluoride (PVDF) membrane with a Trans-Blot SD semidry electrophoretic transfer cell. The membrane was blocked for 15 min with QuickBlock™ Blocking Buffer (P0252, Beyotime, Shanghai, China), then washed with Tris-buffered saline solution containing 0.1% Tween-20 (TBST), three times, 5 min each time. Subsequently, the membrane was incubated with primary antibody at 4 °C overnight, including CYP3A1 (1:100) and PXR (1:500). GAPDH monoclonal antibody was applied as the loading control. The membrane was washed three times in TBST for 10 min per wash and incubated with secondary HRP-conjugated anti-rabbit or anti-mouse IgG antibody for 1 h at room temperature. After adding the enhanced chemiluminescence detection kit (KF005, Affinity Biosciences, Cincinnati, OH, USA) to the membranes, signals were detected using a Bio-Rad ChemiDoc XRS imaging system (Bio-Rad Laboratories, Hercules, CA, USA).

### 2.7. Microsomal Preparation and Enzyme Activity Detection

Liver microsomes were prepared using differential centrifugation as previously described [37,38]. BCA protein analysis kit was used to determine the concentration of liver microsomes. The incubation system (200 μL) consisted of a NADPH regeneration system (Solution A contained 26.1 mM NADP+, 66 mM Glucose-6-phosphate, and 66 mM MgCl_2_, with a volume of 10 μL, Solution B contained 40 U/mL Glucose-6-phosphate dehydrogenase and 5 mM sodium citrate, with a volume of 2 μL, and 137 μL of PBS buffer at pH 7.4), 1 μL of nifedipine (1 mM in acetonitrile), and 50 μL of rat liver microsomes (0.5 mg/mL in the system). The NADPH regeneration system remained unchanged, inactivated liver microsomes with added nifedipine were used as the negative control, and inactivated liver microsomes with added acetonitrile were used as the blank control. Liver microsomes were heat-inactivated at 100 °C for 30 min. At 0 h and 37 °C incubation for 30 min, 50 μL of samples was collected, and 100 μL of precooled acetonitrile was added to stop the reaction. After centrifugation at 4 °C at 1681× *g* for 10 min, 5 μL of supernatant was taken, and 495 μL of 0.1% formic acid water–acetonitrile (1:1, *v*/*v*, containing nitrendipine 50 ng/mL) was added to dilute 100-fold, then vortexed for 2 min, centrifuged at 4 °C and 20,598× *g* for 5 min, and the supernatant was used for HPLC-MS/MS detection.

### 2.8. Primary Hepatocyte Isolation and Induction

Primary rat hepatocytes were isolated from adult male Wistar rats (250–280 g) using a modified two-step collagenase perfusion method [39]. Briefly, the rats were anesthetized by intraperitoneal injection of pentobarbital. The abdomens of the rats were cleaned with alcohol, and the body cavity was opened along the midline. Intestines were moved to expose the portal vein and inferior vena cava (IVC). One suture was placed under the IVC beneath the major branch. The IVC was punctured with an intravenous remained trocar of 20 G and was ligated on it, and the portal vein was cut. The liver was first flushed with saline at a flow rate of 20 mL/min to wash out the blood and then perfused with 200 mL of perfusion buffer (HBSS no Ca^2+^, Mg^2+^, phenol red containing 0.5 mM EDTA, and 25 mM HEPES). Subsequently, the flow rate was reduced to 10 mL/min with 80 mL of digestion buffer that was HBSS containing Ca^2+^, Mg^2+^, phenol red, 50 μg/mL collagenase (C6885, Sigma, St. Louis, MO, USA), and 25 mM HEPES. The digested liver was gently removed from the rat and placed into a Petri dish containing DMEM with 10% FBS. Connective tissues were removed while working in a hood. The livers were sliced into small pieces with sterile tweezers, and the hepatocytes were released into a suspension. The suspension was filtered through 70 μm cell-strainer filters into a 50 mL conical tube and centrifuged at 50× *g* for 3 min at 4 °C. The supernatant was discarded, and the pellet was resuspended in Percoll solution and spun at 100× *g* for 5 min at 4 °C for hepatocyte purification. The supernatant was discarded, and the hepatocytes were washed with DMEM media and then centrifuged at 50× *g* for 3 min at 4 °C. This process was repeated twice. Six-well plates were coated with rat-tail collagen type I (C3867, Sigma, St. Louis, MO, USA) at a final concentration of 0.1 μg/mL. A trypan blue exclusion test was used to determine cell viability, and hepatocytes with a viability of greater than 85% were seeded into six-well plates at 4 × 10^5^ cells per well and cultured in DMEM containing 10% FBS and 1% penicillin–streptomycin solution. Two hours after seeding, the culture medium was replaced with DMEM containing 5% FBS and 1% penicillin–streptomycin solution. After overnight culture, the cells were exposed to a bile-acid-containing medium and incubated for 24 h. RT-PCR was adopted to assess the relative expressions of CYP3A1 and Mdr1a.

### 2.9. Pharmacokinetic Study in Antibiotic-Cocktail-Treated Rats

Twenty-four-week-old SHRs were randomly divided into two groups, the control group (*n* = 8) and the ABx group (*n* = 10). The rats in the ABx group were administered an antibiotic cocktail, including vancomycin (50 mg/kg), neomycin (100 mg/kg), metronidazole (100 mg/kg), and ampicillin (1 mg/mL), for two weeks. The rats in the control group were given an equal volume of saline. After two weeks, the feces were collected and fecal microbial DNA was extracted using a QIAamp^®^ PowerFecal^®^ Pro DNA Kit (51804, QIAGEN, Dusseldorf, Germany) to examine the models that were established successfully. The serum bile acid levels were detected. Three days after antibiotic withdrawal, pharmacokinetic experiments were carried out. The collected blood samples were detected by UPLC-MS/MS after pretreatment.

### 2.10. Blood Biochemistry Test and Histological Assays

Up to 200 μL of rat serum was sent to the laboratory of Wuhan Servicebio Biotechnology Co., Ltd. (Wuhan, China) for the detection of liver function indexes (ALT, AST, and TBA) and kidney function indexes (BUN, Cr, and UA) using an automatic biochemical analyzer. The hepatic lobule, left kidney, and a section of the duodenum, jejunum, and ileum were taken for hematoxylin and eosin (H&E) staining for histopathological examination. The liver tissue was observed under a 5.0× and 20.0× optical microscope, the kidney was observed under a 2.0× and 20.0× optical microscope, and the duodenum, jejunum, and ileum were observed under a 10.0× optical microscope. Specifically, villus height, crypt depth, muscularis thickness, the number of goblet cells, villous epithelium length, and the number of goblet cells per unit length were quantified to assess intestinal pathology.

### 2.11. Statistical Analysis

Statistical analysis between the two groups was evaluated using an unpaired Student’s *t* test with two-tailed distribution or a Mann–Whitney *U* test when data did not coincide with normal distribution. Spearman’s rank correlation test was performed to evaluate the correlations between gut microbiota and pharmacokinetics. All data were expressed as mean ± standard deviation (*SD*). Graphs were generated using GraphPad Prism 8, and statistical analysis was performed using SPSS 25 (IBM, Armonk, NY, USA). *p* < 0.05 represented a statistically significant result.

## 3. Results

### 3.1. Oral Bioavailability of Nifedipine Decreased in SHRs

**Figure 1:**
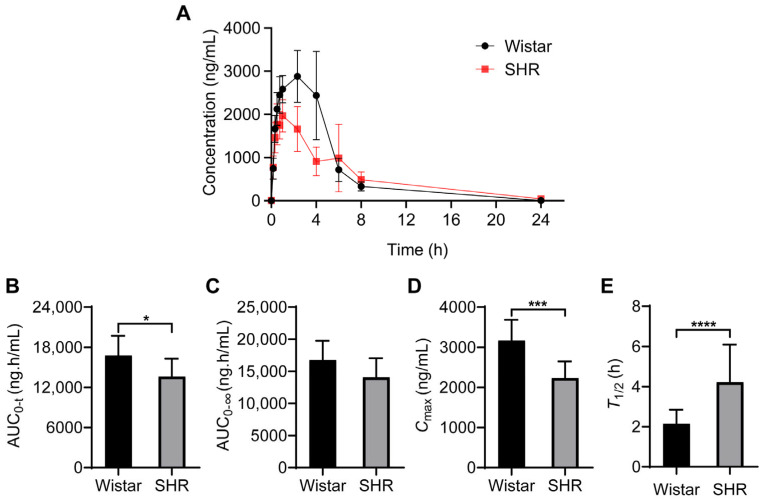
Pharmacokinetic parameters of nifedipine in Wistar rats and SHRs by UPLC-MS/MS. (**A**) Plasma concentration–time curves within 24 h; (**B**–**E**) histogram for statistical analysis of nifedipine pharmacokinetic parameters, AUC_0–t_ (**B**), AUC_0–∞_ (**C**), *C*_max_ (**D**), and *T*_max_ (**E**). Data are expressed as mean ± SD. * *p* < 0.05, *** *p* < 0.001, **** *p* < 0.0001, unpaired *t* test.

The pharmacokinetic parameters of nifedipine were acquired from DAS 2.0 software using a non-compartmental model, including area under the concentration–time curve (AUC), maximum concentration (*C*_max_), mean residence time (MRT), time to reach maximum concentration (*T*_max_), and elimination half-life (*T*_1/2_). We found that the pharmacokinetic characteristics of nifedipine were quite different from each other (Figure 1A). In the Wistar group, the value of AUC_0–t_ was 16,755.62 ± 2963.72 ng·h/mL and *C*_max_ was 3165.00 ± 520.52 ng/mL. In the SHR group, the value of AUC_0–t_ was 13,635.22 ± 2666.00 ng·h/mL and *C*_max_ was 2233.33 ± 417.70 ng/mL (Table 1). Compared with Wistar rats, AUC_0–t_ decreased by 18.62% and *C*_max_ decreased by 29.44% in SHRs (Figure 1B–E, *p* < 0.05). In addition, MRT_0−t_, MRT_0−∞_ and *T*_1/2_ increased by 32.28%, 49.97%, and 96.00% respectively in SHRs (Figure 1B–E, Table 1, *p* < 0.05). Furthermore, we analyzed the intergroup concentrations at each time point, and the results showed that, compared with Wistar rats, the concentrations of nifedipine at 0.75 h, 1.0 h, 2.33 h, and 4 h decreased significantly in SHRs (Table 2).

### 3.2. Gut Microbial Dysbiosis and Bacteroides Enrichment in SHRs

**Figure 2:**
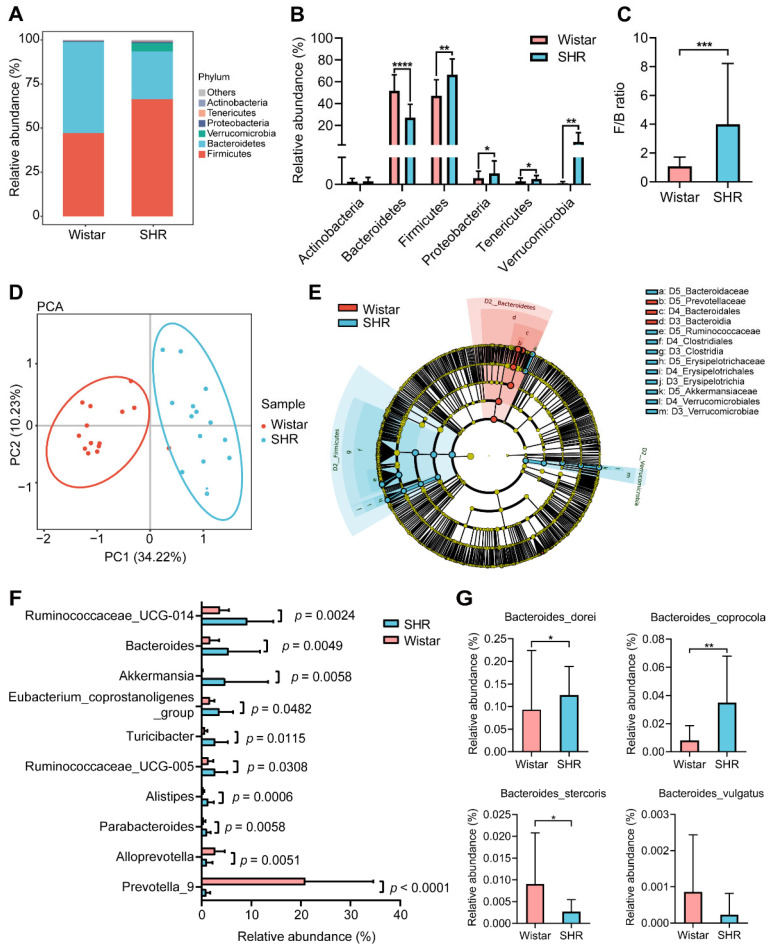
Gut microbial dysbiosis in SHRs. (**A**) Composition of the gut microbiome at phylum taxonomic levels; (**B**) relative abundance of phyla in each group; (**C**) the value of F/B ratio in the two groups; (**D**) principal component analysis (PCA) of gut microbiota diversity among Wistar rats and SHRs; (**E**) LEfSe analysis to find differential taxa based on LDA > 4.0 with Wistar-enriched taxa (blue) and SHR-enriched taxa (red); (**F**) top ten of the most abundant genera with content difference between the two groups; (**G**) relative abundance of *Bacteroides* species, including *Bacteroides_dorei*, *Bacteroides_coprocola*, *Bacteroides_stercoris*, and *Bacteroides_vulgatus.* Data are expressed as mean ± SD. * *p* < 0.05, ** *p* < 0.01, *** *p* < 0.001, **** *p* < 0.0001, unpaired *t* test or Mann–Whitney test.

To investigate the changes in microbial structure between Wistar rats and SHRs, the abundance and diversity of gut microbiota were analyzed using *16S rRNA* gene sequencing. The component and proportion of microbiota changed between groups at the phylum taxonomic levels (Figure 2A). Compared with Wistar, the relative abundance of *Bacteroidetes* in SHRs decreased, while *Firmicutes*, *Proteobacteria*, *Tenericutes*, and *Verrucomicrobia* increased (*p* < 0.05) (Figure 2B). The ratio of *Firmicutes* to *Bacteroidetes* (F/B) was increased in SHRs (*p* < 0.05) compared with Wistar (Figure 2C), indicating disordered gut microbiota in SHRs. PCA analysis indicated that the gut microbial clusters of the two groups were separated distinctly (Figure 2D). LEfSe analysis was implemented to detect differential gut microbial taxonomy between groups. The results showed that at the family level, *Bacteroidaceae*, *Ruminococcaceae*, *Erysipelotrichaceae*, and *Akkermansiaceae* were enriched in SHRs, and *Prevotellaceae* was enriched in Wistar (Figure 2E). The top ten of the most abundant genera with content differences between the two groups are shown in Figure 2F. Except *Alloprevotella* and *Prevotella_9*, the relative abundance of other genera was increased in SHRs, while *Bacteroides* was the second richest genera (*p* = 0.0049). Analyzing the abundance of *Bacteroides* species indicated that *Bacteroides dorei* and *Bacteroides coprocola* were increased and *Bacteroides stercoris* was decreased, yet other strains had no obvious difference (e.g., *Bacteroides vulgatus*) (Figure 2G).

### 3.3. Association between Gut Microbiota and Pharmacokinetic Parameters

**Figure 3:**
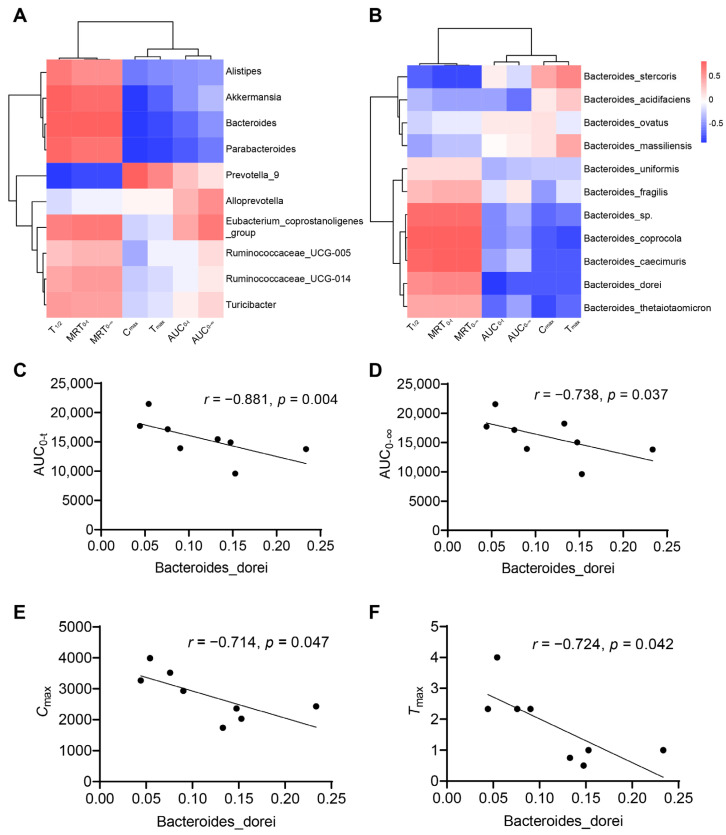
Correlations between gut microbiota and pharmacokinetic parameters. (**A**) Heatmap of correlations between top ten of the most abundant genera with content difference and pharmacokinetic parameters; (**B**) heatmap of correlations between the species in *Bacteroides* and pharmacokinetic parameters; (**C**–**F**) *Bacteroides dorei* is negatively correlated with AUC_0–t_ (*r* = −0.881, *p* = 0.004), AUC_0–∞_ (*r* = −0.738, *p* = 0.037), *C*_max_ (*r* = −0.714, *p* = 0.047), and *T*_max_ (*r* = −0.724, *p* = 0.042). Data are expressed as mean ± SD, unpaired *t* test or Mann–Whitney test.

Considering that the changes in pharmacokinetic characters were in parallel with the dysbiosis in gut microbiota, we evaluated whether the specific species was correlated with pharmacokinetic parameters (AUC_0–t_, AUC_0–∞_, *C*_max_, *T*_max_, MRT_0–t_, MRT_0–∞_, *T*_1/2_) in the two groups. Spearman’s correlation test was used to assess the relationship between the genera in Figure 2F and the pharmacokinetic parameters of nifedipine. The result showed that the relative abundances of *Akkermansia, Bacteroides,* and *Parabacteroides* were negatively correlated with *C*_max_ (*p* < 0.05), and *Prevotella_9* was positively correlated with *C*_max_ (*p* < 0.05) (Figure 3A). *Bacteroides* was also positively correlated with MRT_0–t_, MRT_0–∞_, and *T*_1/2_ with significance (Figure 3A). We further analyzed the correlation between the species in *Bacteroides* and the pharmacokinetic parameters (Figure 3B). We found *Bacteroides dorei* was negatively correlated with AUC_0–t_ (*r* = −0.881, *p* = 0.004), AUC_0–∞_ (*r* = −0.738, *p* = 0.037), *C*_max_ (*r* = −0.714, *p* = 0.047), and *T*_max_ (*r* = −0.724, *p* = 0.042) (Figure 3C–F).

### 3.4. Perturbations of the Microbiota Altered the BA Profiles in SHRs

**Figure 4:**
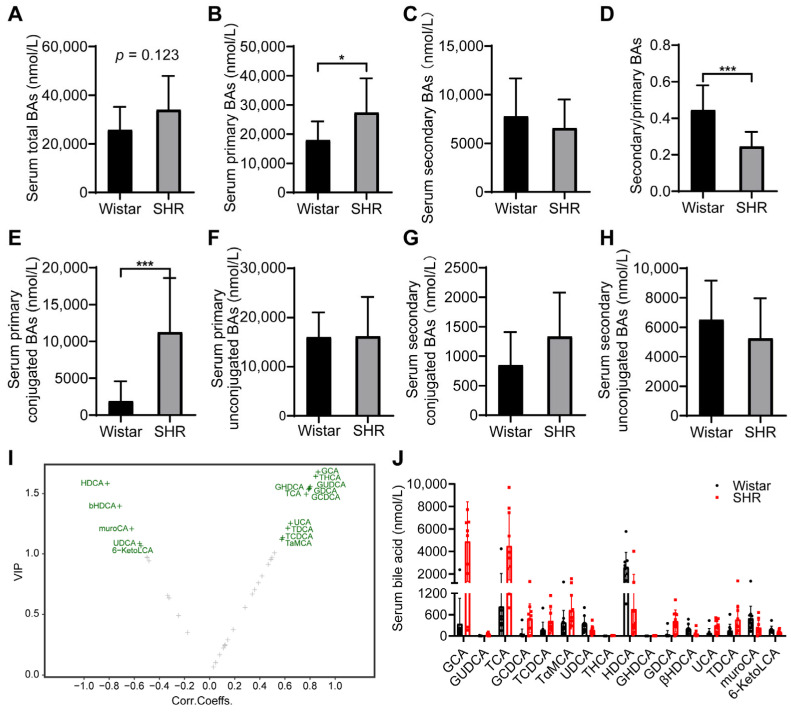
Effect of intestinal dysbacteriosis on serum BA profiles. (**A**) Concentrations of total BAs; (**B**) concentrations of primary BAs; (**C**) concentrations of secondary BAs; (**D**) ratio of secondary to primary BAs; (**E**,**F**) concentrations of primary conjugated and unconjugated BAs; (**G**,**H**) concentrations of secondary conjugated and unconjugated BAs; (**I**) volcano plot of 16 differential BAs by variable importance in projection (VIP) of OPLAS-DA model; (**J**) concentrations of 16 differential BAs in two groups. Data are expressed as mean ± SD. * *p* < 0.05, *** *p* < 0.001, unpaired *t* test or Mann–Whitney test.

To investigate whether the changes in microbiota affected the BA profiles, UPLC/MS/MS was applied to measure the concentration of BAs in serum. The content of serum total BAs was slightly higher in the SHR group (*p* = 0.123, Figure 4A) compared to in the Wistar rat group. A significant increase in the content of primary BAs accompanied by a lower value of secondary to primary BA ratio in SHRs was observed (Figure 4B–D). Furthermore, the SHR group had a higher content of primary conjugated BAs (Figure 4E). The content of primary unconjugated BAs, secondary conjugated BAs, and secondary unconjugated BAs did not differ between the two groups (Figure 4F–H). Sixteen differential BAs were selected by orthogonal partial least squares discriminant analysis (OPLS-DA) (Figure 4I), including seven primary BAs (GCA, TCA, GUDCA, GCDCA, TCDCA, TαMCA, and UDCA) and nine kinds of secondary BAs (THCA, HDCA, GHDCA, GDCA, βHDCA, UCA, TDCA, muroCA, and 6-KetoLCA) (Figure 4J, Table 3).

### 3.5. Gut Microbiota Altered the Expression and Enzyme Activity of CYP3A1

**Figure 5:**
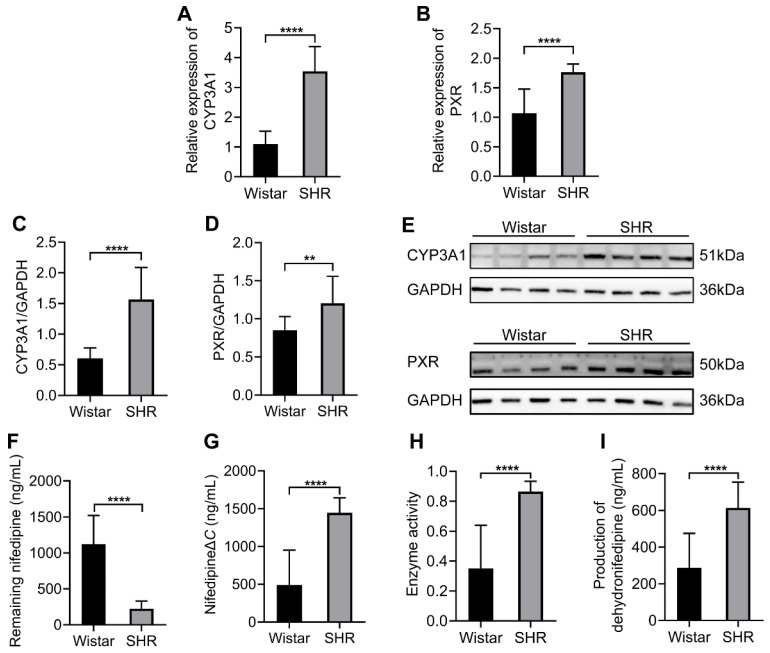
Comparison of the expression of CYP3A1 and PXR and the enzyme activity of CYP3A1 between the two groups in the liver. (**A**,**B**) The expressions of CYP3A1 and PXR in mRNA level; (**C**,**D**) integrated density to quantify the expression of CYP3A1 and PXR in protein levels; (**E**) Western blotting of CYP3A1 and PXR; (**F**) remaining nifedipine in the co-incubation system after 30 min; (**G**) reduction in nifedipine; (**H**) the enzyme activity of CYP3A1, which is the ratio of reduction in nifedipine to the total amount of it; (**I**) Production of dehydronifedipine. Data are expressed as mean ± SD. ** *p* < 0.01, **** *p* < 0.0001, unpaired *t* test or Mann–Whitney test.

To test the expressions of CYP3A1 and PXR under gut microbial dysbiosis in SHRs, quantitative real-time PCR and Western blot were performed to examine the hepatic expression of CYP3A1 and PXR, and the results showed that the expressions of CYP3A1 and PXR were upregulated both in mRNA and protein levels (*p* < 0.05) in the SHRs (Figure 5A–E). The enzyme activity of CYP3A1 in liver microsomes was determined using HPLC-MS/MS. After the co-incubation of nifedipine and liver microsomes for 30 min, the residual amount of nifedipine was 225.3 ± 105.9 ng/mL in the SHR group and 1120.0 ± 400.8 ng/mL in the Wistar group (Figure 5F). The reduction in nifedipine was 1446.0 ± 197.6 ng/mL in the SHR group and 491.3 ± 461.8 ng/mL in the Wistar group (Figure 5G). The enzyme activity of CYP3A1 in the SHR group was 2.45 times higher than that in the Wistar group (Figure 5H). The production of dehydronifedipine was 614.1 ± 140.0 ng/mL in the SHR group and 286.8 ± 187.9 ng/mL in the Wistar group (Figure 5I). Because the liver played a primary role in the metabolic process of nifedipine, the upregulation of CYP3A1 and PXR expression and increased enzyme activity of CYP3A1 in SHRs may contribute to the reduced bioavailability of nifedipine in SHRs.

### 3.6. Correlation among Microbiota, Bile Acids, and Pharmacokinetic Parameters

**Figure 6:**
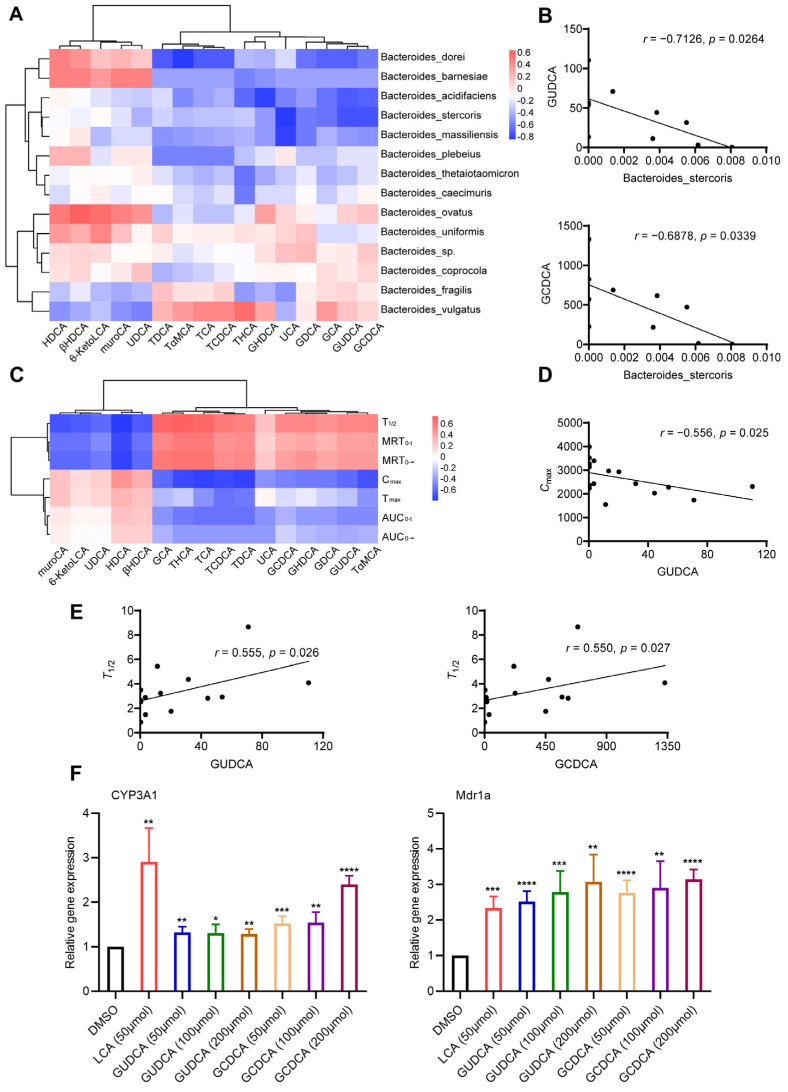
Correlation among gut microbiota, serum BAs, and pharmacokinetic parameters. (**A**) Spearman correlation analysis of *Bacteroides* species and 16 differential BAs; (**B**) *Bacteroides stercoris* is negatively correlated with GUDCA (*r* = −0.7126, *p* = 0.0264) and GCDCA (*r* = −0.6878, *p* = 0.0339); (**C**) Spearman correlation analysis of 16 differential BAs and pharmacokinetic parameters; (**D**) GUDCA is negatively correlated with *C*_max_ (*r* = −0.556, *p* = 0.025); (**E**) GUDCA (*r* = −0.555, *p* = 0.026) or GCDCA (*r* = −0.550, *p* = 0.027) is positively correlated with *T*_1/2_; (**F**) relative expressions of CYP3A1 and Mdr1a in hepatocytes after being treated with GUCDA and GCDCA. DMSO-treated as a negative control and LCA-treated as a positive control. Data are expressed as mean ± SD. * *p* < 0.05, ** *p* < 0.01, *** *p* < 0.001, **** *p* < 0.0001, unpaired *t* test or Mann–Whitney test.

Considering that bile acid may be the signaling molecule of intestinal microbiota influencing pharmacokinetics, a Spearman’s correlation test was used to explore the relationship between intestinal microbiota and specific bile acids and between bile acids and pharmacokinetic parameters. The results showed that the relative abundance of *Bacteroides stercoris* had negative correlations with the contents of GUDCA and GCDCA (*p* < 0.05), and the correlation coefficients were −0.7126 and −0.6878, respectively (Figure 6A,B). A heatmap of the correlations between bile acids and pharmacokinetic parameters is shown in Figure 6C. The concentration of GUDCA was negatively correlated with *C*_max_ (*r* = −0.556, *p* = 0.025) and positively correlated with *T*_1/2_ (*r* = 0.555, *p* = 0.026) (Figure 6D,E). GCDCA was also positively correlated with *T*_1/2_ (*r* = 0.550, *p* = 0.027) (Figure 6E). Primary hepatocytes were considered to be an ideal model for an induction experiment in vitro. To explore the interaction between bile acids and PXR, primary rat hepatocytes were isolated and treated with different concentrations of GUDCA and GCDCA. The secondary bile acid LCA was an efficacious activator of PXR and included as a positive control. The relative expressions of CYP3A1 and Mdr1a in the GUDCA-/GCDCA-treated groups were higher (*p* < 0.05) than those in the DMSO-treated group (Figure 6F).

### 3.7. Using the Antibiotic-Cocktail-Treated Rats to Confirm That Gut Microbiota Are Involved in the Metabolism of Nifedipine

**Figure 7:**
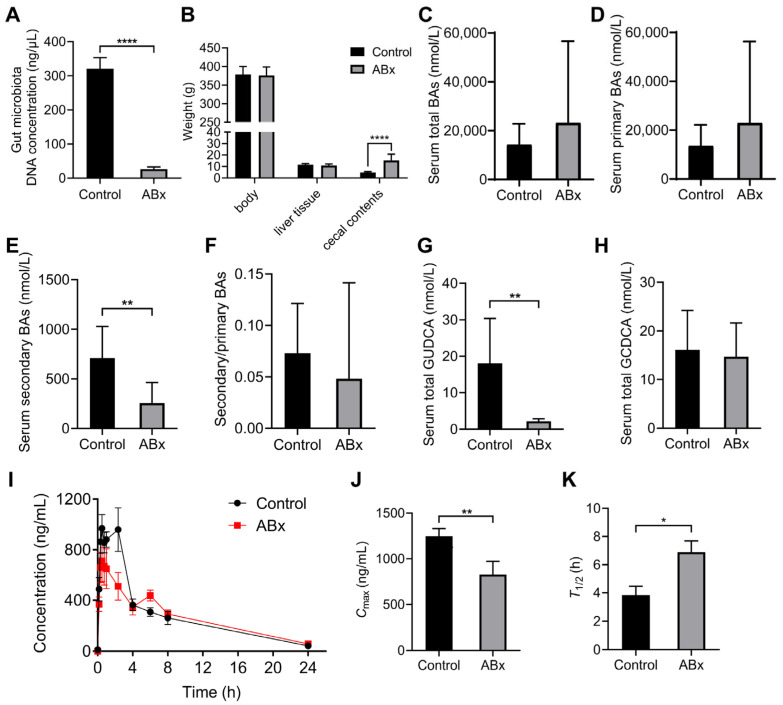
Validation of microbial biotransformation of nifedipine by antibiotic-cocktail-treated rats. (**A**) The concentration of gut microbial DNA in stool samples; (**B**) the weight of the body, liver tissues, and cecal contents; (**C**–**F**) analysis of serum BAs profiles; (**G**) the content of serum total GUDCA; (**H**) the content of serum total GCDCA; (**I**) plasma concentration–time curves within 24 h in antibiotic-cocktail-treated rats; (**J**,**K**) comparison of the values of *C*_max_ and *T*_1/2_ between groups. Data are expressed as mean ± SD. * *p* < 0.05, ** *p* < 0.01, **** *p* < 0.0001, unpaired *t* test or Mann–Whitney test.

Germ-free or antibiotic-cocktail-treated models are usually used to study the relationship between gut microbiota and diseases or the metabolism of drugs [40]; we adopted an antibiotic cocktail to deplete the gut microbiota in SHRs. We extracted the gut microbial DNA from fecal samples and detected concentration; the results showed that compared with the control group, the value was decreased by 91.7% in the ABx group (Figure 7A). The weight of cecal contents in the ABx groups was higher than that in control groups (*p* < 0.0001), while the body weight and liver tissue weight showed no difference (Figure 7B). After the model was constructed, we measured the serum BAs; the results showed that the contents of total BAs and primary BAs had no significant difference in the ABx group and control group (Figure 7C,D), while secondary BAs were significantly decreased in the ABx group (Figure 7E). Moreover, total GUDCA was lower in the ABx group than in the control group (*p* < 0.01) (Figure 7G). We further performed the pharmacokinetic study in antibiotic-cocktail-treated rats. The pharmacokinetic curves are shown in Figure 7I. Compared with the control group, the *C*_max_ was decreased (*p* < 0.01) and the *T*_1/2_ was increased (*p* < 0.05) in the ABx group (Figure 7J,K).

### 3.8. Effect of Liver and Renal Functions on Drug Metabolism and Intestinal Functions on Drug Absorption

**Figure 8:**
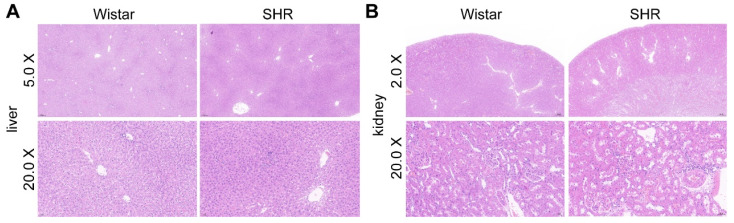
H&E staining of the liver (**A**) and the kidney (**B**).

**Figure 9:**
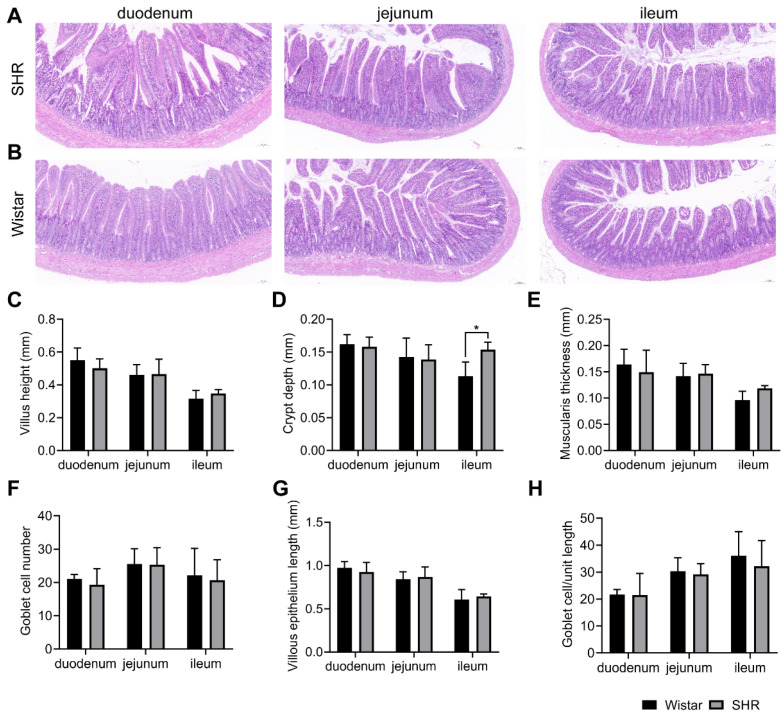
Gut morphology and measurement analysis of H&E staining in Wistar rats and SHRs. (**A**,**B**) H&E staining of the duodenum, jejunum, and ileum (10×) in SHRs and Wistar rats; (**C**–**H**) measurement of intestinal pathological parameters, including villus height (**C**), crypt depth (**D**), muscularis thickness (**E**), goblet cell number (**F**), villous epithelium length (**G**), and goblet cell/unit length (**H**). Data are expressed as mean ± SD. * *p* < 0.05, unpaired *t* test or Mann–Whitney test.

The liver and kidney functions under hypertension are the basic factors affecting drug metabolism. To exclude the possible influences of liver and kidney functions on pharmacokinetics, the serum biochemical indexes of the SHR group and Wistar group were detected. There were no pathological changes in the H&E staining results of the liver and kidney (Figure 8A,B). No abnormalities in liver function indexes (ALT, AST, TBA) and kidney function indexes (BUN, Cr, UA) were observed between the two groups (Table 4). By observing the pharmacokinetic curves in Figure 1A, we found that the process of drug absorption differed between the two groups. The intestinal histological and measurement analysis showed that there were no obvious pathological changes in the intestinal tissues of the two groups (Figure 9), but the crypt depth of the ileum in SHRs was greater than that of the Wistar rats (Figure 9D).

## 4. Discussion

The pharmacokinetics of nifedipine exhibit significant variability, and its antihypertensive efficacy demonstrates considerable variation among individuals at the clinical level. The gut microbiome harbors diverse catalytic enzymes and is regarded as a ‘metabolic organ’ capable of influencing host metabolism [41,42]. Previous studies have proven that gut microbiota can mediate the metabolism of many oral medications [41]. During oral administration, the drug is absorbed into blood circulation through the gastrointestinal tract and contacts many thousands of intestinal microbial species [43,44]. Enzymes synthesized by microbiota can modify the structure of a drug and alter its bioavailability [45,46]. It has been discovered that the gut microbiota plays a role in the pharmacokinetics of antihypertensive drugs [47,48]. The gut microbiota may affect the bioavailability of antihypertensive drugs through a variety of ways, including bacterial metabolism, bacterial transport, and regulation of drug metabolic enzymes and intestinal transports [47]. In this study, we explored how the gut microbiota affects the pharmacokinetics of nifedipine and influences its bioavailability. By conducting pharmacokinetic experiments and analyzing the relative abundance of microbiota, the levels of bile acids, and the enzyme activity of CYP3A1, we observed that the gut microbiota directly metabolizes nifedipine or regulates the enzyme activity of CYP3A1 by bile acids.

As we aimed to investigate the role of microbiota in the variability of nifedipine’s pharmacokinetics, animal models of spontaneous hypertension (SHRs) and normotension (Wistar rats) were utilized to evaluate the pharmacokinetics. The findings revealed a significant decrease in the bioavailability of nifedipine in SHRs compared to Wistar rats, indicating a reduction in nifedipine metabolism. Subsequently, we used 16S rRNA gene sequencing to detect the composition and relative abundance of microbial taxonomies. We found that in the SHR group, the ratio of *Firmicutes* to *Bacteroidetes* (F/B ratio) increased, which is a potential biomarker of gut microbial dysbiosis [49,50]. Our results are consistent with those of previous studies. For example, Li et al. [23] found that, compared with normotensive WKY rats, the F/B ratio of SHRs is increased (*p* < 0.05). In addition, Santisteban et al. [27] investigated whether the changes in the F/B ratio were associated with the development of hypertension, and no difference was found in the F/B ratio between prehypertensive SHRs and age-matched WKY rats, indicating that as blood pressure increased, the gut microbiota was disrupted. Overall, a remarkable gut microbial dysbiosis occurred in SHRs.

To explore the direct biotransformation of nifedipine by microbiota, we conjointly analyzed the pharmacokinetic parameters and 16S rRNA gene sequencing data. We found that several genera were related to the pharmacokinetics of nifedipine, in which *Bacteroides* was negatively correlated with *C*_max_ and positively correlated with MRT_0–t_, MRT_0–∞_, and *T*_1/2_. Furthermore, Spearman correlation analysis showed that the relative abundance of *Bacteroides dorei* was negatively correlated with the values of AUC_0–t_, AUC_0–∞_, *C*_max_, and *T*_max_. This indicated that the dysbiosis of microbiota under hypertension may play a role in the pharmacokinetic difference in nifedipine, and *Bacteroides dorei* may have the enzyme activity of metabolizing nifedipine directly. Previous studies have explored the effect of gut microbiota on the pharmacokinetics of nifedipine. Zhang et al. [32] revealed that the metabolic activity of intestinal flora decreased with the bioavailability of nifedipine being increased in a plateau hypoxia environment [32], but this study failed to identify the specific bacterial strains that contributed to the changes in pharmacokinetic characteristics. Kato et al. [51] reported that oral ingestion of the probiotic *Lactobacillus casei* can improve the bioavailability of nifedipine, and treatment with *L. casei* can increase the absorption of nifedipine by decreasing intestinal CYP3A activity to increase intestinal availability. Overall, the microbial metabolism of nifedipine may provide a potential explanation for its pharmacokinetic variability. Besides hypertension, which is focused on in this study, NAFLD is one of the most common liver diseases and can develop into NASH, leading to hepatic pathological changes and lower liver function [52]. Guo et al. constructed a mice model with NASH and proved that gut microbiota and host CYP450s co-contribute to pharmacokinetic variability under NASH [6].

It is known that gut microbiota can influence drugs through indirect ways, including the regulation of host gene expression. Intestinal microbiota can upregulate the expression of CYP enzymes and nuclear receptor PXR [53]. PXR is a key regulator of the exogenous induction of CYP3A4 expression [54]. BAs as endogenous ligands of the nuclear receptor PXR have been widely studied. LCA, a secondary bile acid, was derived from gut microbiota and has been proven to activate PXR and induce CYP3A [55]. In this study, whether bile acid plays a role should be explored. In the analysis of the BA profiles, the contents of primary BAs and primary conjugated BAs were significantly elevated in the serum of SHRs compared with Wistar rats. Sixteen differential BAs were screened by the OPLAS-DA model, which includes six kinds of primary conjugated BAs, including GUDCA, GCA, GCDCA, TCA, TCDCA, and TαMCA. In general, the disturbance of gut microbiota changed the serum BA profiles.

Nifedipine is primarily oxidized by human CYP3A4 and rat CYP3A1 [3,56]. We found that both at mRNA and protein levels, the expression of CYP3A1 in SHRs was increased; meanwhile, the expression of PXR also increased. The enzyme activity of CYP3A1 was proved to be increased in SHRs by the experiment on liver microsomal metabolism. The upregulated expression and increased activity of CYP3A1 in SHRs promoted the hepatic metabolism of nifedipine. Therefore, the disorder of intestinal microbiota influenced the CYP3A1 enzyme and affected the bioavailability of nifedipine. Moreover, the CYP3A subfamily metabolized ~30% of clinically used drugs; other substrates would also be influenced in hypertension.

Considering that gut microbiota may affect the activity of the CYP3A1 enzyme through BAs, we analyzed the correlations among the species of *Bacteroides*, the differential BAs, and pharmacokinetic parameters. The results showed that *Bacteroides stercoris* may modulate the contents of GUDCA and GCDCA to affect the pharmacokinetics. Further, GUDCA and GCDCA were proven to induce CYP3A1 in primary rat hepatocytes. Bile acids that have been reported to activate PXR, including LCA, CDCA, DCA, and CA, showed no significant difference between the two groups in our research. The existing studies on the BA activation of PXR mainly focus on unconjugated BAs, and the rank order of induced affinity is 3-keto-LCA > LCA > CDCA, DCA > CA [55,57]. UDCA and TUDCA have also been reported to activate PXR and induce CYP3A4 [58]. Considering that PXR is a promiscuous nuclear receptor, there is species specificity in its ligand binding pocket [59]; the amino acid sequences of PXR’s LBD among different species share only 75~80% of their identity [60], suggesting that inter-species variation in PXR ligand specificity exists [19]. Moreover, other metabolites derived from the microbiota and influenced drug metabolism have been reported. Short-chain fatty acids (SCFAs), derivative metabolites of intestinal flora, have also been reported to improve the metabolic activity of liver organoids, including the expression of CYP3A4 [61]. Indole-3-propionic acid (IPA), a metabolite of tryptophan metabolized by bacteria, can also activate PXR [62], and its effect on CYP3A enzyme activity remains to be explored. In addition, a specific bacterial strain that influenced the enzyme activity of CYP3A has been reported. For example, Liu J et al. found that *Lactobacillus rhamnosus* induced CYP3A activity [63].

To validate the impact of gut microbiota on the pharmacokinetics, we used antibiotic-cocktail-treated rats in pharmacokinetic experiments on nifedipine. The results showed that the *C*_max_ decreased and the *T*_1/2_ increased in the ABx group compared with the control group, indicating that the depletion of microbiota reduced the extent of drug absorption and extended the time of drug metabolism. We also detected the serum BA profiles and we found that the concentrations of secondary BAs decreased in the ABx group, which could be explained by the impaired process of gut-microbiota-mediated transformation of primary bile acid into secondary bile acid. Moreover, the content of GUDCA was decreased after antibiotic cocktail treatment, while the content of GUDCA, as a primary conjugated bile acid, should theoretically increase or remain unchanged in the ABx group. An increase in GCDCA was observed by Yang et al. in antibiotic-treated Wistar rats, while no difference occurred in the content of GUDCA [13]. The discrepancies between our results and the previous study could be explained by the different rat strains and the relatively small sample size, as well as the sensitivity of detection. In summary, antibiotic-cocktail-treated rats verified the effect of the gut microbiota on the BA profiles and the pharmacokinetics of nifedipine.

## 5. Limitations of This Study

Although the potential involvement of *Bacteroides dorei* and *Bacteroides stercoris* in the pharmacokinetics of nifedipine has been identified, further confirmation requires the establishment of animal models specifically targeting the knockout of these bacteria. In addition, given the intricate nature of the gut microbiota and bile acids, as well as the disparities between experimental models and humans, it is necessary to validate whether the results of this study can be extrapolated to humans. Notably, individual differences in gut microbiota exist in hypertensive patients, and with the progress of hypertension, the gut microbiota of each patient will also change, which is a more complex problem and needs to be explored in the clinic.

## 6. Conclusions

Our study reported a reduction in the bioavailability of nifedipine in SHRs and investigated the underlying mechanism involving the gut microbiota. This mechanism includes the direct metabolism of nifedipine by bacterial enzymes or the indirect modulation of its metabolism through the upregulation of CYP3A1 activity. The gut microbiota plays a role in the progress of hypertension, and gut microbial disorder has been observed in hypertensive patients [64]. Therefore, considering the impact of the gut microbiota on nifedipine’s pharmacokinetics, our findings offer a microbial-oriented approach to address the interindividual variability in the clinical response to antihypertensive drugs. Further research can focus on how to translate the results of animal experiments into clinical practice. We should also explore how to reshape the gut microbiota to improve the bioavailability of drugs.

## Figures and Tables

**Table 1:** Pharmacokinetic parameters of nifedipine.

Parameter	Unit	Wistar	SHR
AUC_0–t_	ng·h/mL	16,755.62 ± 2963.72	13,635.22 ± 2666.00 *
AUC_0–∞_	ng·h/mL	16,780.48 ± 2977.64	14,073.07 ± 2974.92
*C*_max_	ng/mL	3165.00 ± 520.52	2233.33 ± 417.70 ***
*T*_max_	h	2.21 ± 0.94	1.86 ± 1.74
MRT_0–t_	h	3.85 ± 0.34	5.09 ± 1.13 ***
MRT_0–∞_	h	3.88 ± 0.36	5.82 ± 2.48 ***
*T*_1/2_	h	2.15 ± 0.70	4.22 ± 1.87 ****

The data are expressed as the mean ± standard deviation (*SD*), *n* = 8–9/group. * *p* < 0.05, *** *p* < 0.001, **** *p* < 0.0001.

**Table 2:** The concentration of nifedipine at each time point.

Time (h)	Wistar	SHR
0.167	744.10 ± 245.40	771.80 ± 267.10
0.33	1666.00 ± 311.60	1462.00 ± 348.90
0.5	2125.00 ± 380.50	1771.00 ± 475.50
0.75	2451.00 ± 422.50	1748.00 ± 317.00 **
1	2586.00 ± 318.90	1969.00 ± 373.00 **
2.33	2881.00 ± 600.70	1661.00 ± 521.70 ***
4	2440.00 ± 1024.00	910.80 ± 331.80 ***
6	717.00 ± 272.70	988.90 ± 782.10
8	330.20 ± 106.40	488.90 ± 177.30 *
24	6.11 ± 3.69	44.94 ± 67.24 **

The data are expressed as the mean ± standard deviation (*SD*), *n* = 8–9/group. * *p* < 0.05, ** *p* < 0.01, *** *p* < 0.001.

**Table 3:** The potential biomarkers of BA profile. Metabolites were ranked from large to small by the value of fold change (FC) (*n* = 10/group).

Metabolite	Class	Uni_P	Uni_FDR	FC	log2FC	OPLSDA_VIP
GUDCA	Primary BAs	0.0030	0.0204	113.81	6.83	1.5485
GHDCA	Secondary BAs	0.0032	0.0204	50.33	5.65	1.5352
GCA	Primary BAs	2.06 × 10^−4^	0.0086	47.29	5.56	1.6696
GDCA	Secondary BAs	0.0039	0.0204	46.69	5.54	1.5250
GCDCA	Primary BAs	0.0089	0.0313	31.92	5.00	1.4863
THCA	Secondary BAs	0.0010	0.0139	27.41	4.78	1.6344
TCA	Primary BAs	4.87 × 10^−4^	0.0102	8.84	3.14	1.5364
UCA	Secondary BAs	0.0115	0.0371	7.75	2.95	1.2438
TDCA	Secondary BAs	0.0089	0.0313	3.32	1.73	1.2045
TCDCA	Primary BAs	0.0089	0.0313	2.63	1.40	1.1256
TαMCA	Primary BAs	0.0355	0.0784	2.34	1.23	1.1112
6-KetoLCA	Secondary BAs	0.0232	0.0610	0.62	−0.68	1.0576
UDCA	Primary BAs	0.0426	0.0895	0.50	−1.01	1.0772
muroCA	Secondary BAs	0.0288	0.0712	0.42	−1.24	1.1990
βHDCA	Secondary BAs	0.0089	0.0313	0.15	−2.70	1.3869
HDCA	Secondary BAs	0.0015	0.0158	0.12	−3.06	1.5747

**Table 4:** Serum biochemical indexes of liver function and kidney function.

Parameter	Unit	Wistar	SHR
AST	U/L	432.90 ± 182.80	498.20 ± 66.04
ALT	U/L	99.73 ± 45.88	90.33 ± 56.11
TBA	μmol/L	6.24 ± 2.35	8.30 ± 3.43
BUN	mmol/L	20.31 ± 2.77	21.67 ± 2.06
Cr	μmol/L	58.54 ± 10.28	52.78 ± 8.35
UA	μmol/L	142.20 ± 57.29	173.40 ± 72.93

The data are expressed as the mean ± standard deviation (*SD*), *n* = 8–9/group. ALT: alanine aminotransferase; AST: aspartate aminotransferase; TBA: total bile acid; BUN: blood urea nitrogen; Cr: creatinine; UA: uric acid.

## Data Availability

Metadata of the 16S rRNA gene sequencing have been deposited in the NCBI SRA database with the BioProject accession number PRJNA792311.

## Supplementary Materials

The following supporting information can be downloaded at: https://www.mdpi.com/article/10.3390/pharmaceutics15082085/s1, Figure S1: Product ion spectrum of (A) nifedipine, (C) nitrendipine and (E) dehydronifedipine; HPLC chromatographs of (B) nifedipine, (D) nitrendipine and (F) dehydronifedipine; Table S1: Primer sequences used for RT-PCR.

## Abbreviations

ALT, alanine aminotransferase; AST, aspartate aminotransferase; AUC, area under the concentration-time curve; BAs, bile acids; BSH, bile salt hydrolases; BUN, blood urea nitrogen; *C*_max_, maximum concentration; Cr, creatinine; Ct, cycle threshold; CYP450, cytochrome P450; D-NF, dehydronifedipine; F/B, Firmicutes to Bacteroidetes; FXR, farnesoid X receptor; GCDCA, glycochenodeoxycholic acid; GUDCA, glycoursodeoxycholic acid; H&E, Hematoxylin and eosin; Hnf4α, hepatocyte nuclear factor 4α; HSDH, hydroxysteroid dehydrogenases; IPA, indole-3-propionic acid; IVC, inferior vena cava; LEfSe, line discriminant analysis effect size; MRT, mean residence time; NF, nifedipine; OPLSDA, orthogonal partial least-squares discrimination analysis; OTUs, operational taxonomic units; PVDF, polyvinylidene fluoride; PXR, pregnane X receptor; RXR, retinoid X receptor; SBP, systolic blood pressure; SCFA, short-chain fatty acid; SDS-PAGE, sodium dodecyl sulfate polyacrylamide gel; SHR, spontaneously hypertensive rat; TBA, total bile acid; TGR5, G-protein-coupled bile acid receptor; *T*_max_, time to reach maximum concentration; *T*_1/2_, elimination half-life; UA, uric acid; VDR, vitamin D3 receptor; VIP, variable importance in projection.

## References

[1] Sorkin E.M., Clissold S.P., Brogden R.N. (1985). Nifedipine A Review of Its Pharmacodynamic and Pharmacokinetic Properties, and Therapeutic Efficacy, in Ischaemic Heart Disease, Hypertension and Related Cardiovascular Disorders. Drug Eval..

[2] Nader A.M., Quinney S.K., Fadda H.M., Foster D.R. (2016). Effect of Gastric Fluid Volume on the In Vitro Dissolution and In Vivo Absorption of BCS Class II Drugs: A Case Study with Nifedipine. AAPS J..

[3] Wang H.J., Lu C.K., Chen W.C., Chen A.C., Ueng Y.F. (2019). Shenmai-Yin decreased the clearance of nifedipine in rats: The involvement of time-dependent inhibition of nifedipine oxidation. J. Food Drug Anal..

[4] Krecic-Shepard M.E., Park K., Barnas C., Slimko J., Kerwin D.R., Schwartz J.B. (2000). Race and sex influence clearance of nifedipine: Results of a population study. Clin. Pharmacol. Ther..

[5] Littler W.A. (1990). Control of blood pressure in hypertensive patients with felodipine extended release or nifedipine retard. Br. J. Clin. Pharmacol..

[6] Guo J., Xu Y., Chen L.-J., Zhang S.-X., Liou Y.-L., Chen X.-P., Tan Z.-R., Zhou H.-H., Zhang W., Chen Y. (2021). Gut microbiota and host Cyp450s co-contribute to pharmacokinetic variability in mice with non-alcoholic steatohepatitis: Effects vary from drug to drug. J. Adv. Res..

[7] Marques F.Z., Mackay C.R., Kaye D.M. (2018). Beyond gut feelings: How the gut microbiota regulates blood pressure. Nat. Rev. Cardiol..

[8] Weersma R.K., Zhernakova A., Fu J. (2020). Interaction between drugs and the gut microbiome. Gut.

[9] Javdan B., Lopez J.G., Chankhamjon P., Lee Y.J., Hull R., Wu Q., Wang X., Chatterjee S., Donia M.S. (2020). Personalized Mapping of Drug Metabolism by the Human Gut Microbiome. Cell.

[10] Haiser H.J., Gootenberg D.B., Chatman K., Sirasani G., Balskus E.P., Turnbaugh P.J. (2013). Predicting and manipulating cardiac drug inactivation by the human gut bacterium *Eggerthella lenta*. Science.

[11] Zimmermann M., Zimmermann-Kogadeeva M., Wegmann R., Goodman A.L. (2019). Mapping human microbiome drug metabolism by gut bacteria and their genes. Nature.

[12] Meinl W., Sczesny S., Brigelius-Flohé R., Blaut M., Glatt H. (2009). Impact of gut microbiota on intestinal and hepatic levels of phase 2 xenobiotic-metabolizing enzymes in the rat. Drug Metab. Dispos. Biol. Fate Chem..

[13] Yang H., Zhang Y., Zhou R., Wu T., Zhu P., Liu Y., Zhou J., Xiong Y., Xiong Y., Zhou H. (2023). Antibiotics-induced depletion of rat microbiota induces changes in the expression of host drug-processing genes and pharmacokinetic behaviors of CYPs probe drugs. Drug Metab. Dispos. Biol. Fate Chem..

[14] Zhou J., Zhang R., Guo P., Li P., Huang X., Wei Y., Yang C., Zhou J., Yang T., Liu Y. (2022). Effects of intestinal microbiota on pharmacokinetics of cyclosporine a in rats. Front. Microbiol..

[15] Kaddurah-Daouk R., Baillie R.A., Zhu H., Zeng Z.B., Wiest M.M., Nguyen U.T., Wojnoonski K., Watkins S.M., Trupp M., Krauss R.M. (2011). Enteric microbiome metabolites correlate with response to simvastatin treatment. PLoS ONE.

[16] Wahlström A., Sayin S.I., Marschall H.U., Bäckhed F. (2016). Intestinal Crosstalk between Bile Acids and Microbiota and Its Impact on Host Metabolism. Cell Metab..

[17] de Aguiar Vallim T.Q., Tarling E.J., Edwards P.A. (2013). Pleiotropic roles of bile acids in metabolism. Cell Metab..

[18] Schaap F.G., Trauner M., Jansen P.L. (2014). Bile acid receptors as targets for drug development. Nat. Rev. Gastroenterol. Hepatol..

[19] Jonker J.W., Liddle C., Downes M. (2012). FXR and PXR: Potential therapeutic targets in cholestasis. J. Steroid Biochem. Mol. Biol..

[20] Mills K.T., Stefanescu A., He J. (2020). The global epidemiology of hypertension. Nat. Rev. Nephrol..

[21] Lu Y., Wang P., Zhou T., Lu J., Spatz E.S., Nasir K., Jiang L., Krumholz H.M. (2018). Comparison of Prevalence, Awareness, Treatment, and Control of Cardiovascular Risk Factors in China and the United States. J. Am. Heart Assoc..

[22] Lu J., Lu Y., Wang X., Li X., Linderman G.C., Wu C., Cheng X., Mu L., Zhang H., Liu J. (2017). Prevalence, awareness, treatment, and control of hypertension in China: Data from 1·7 million adults in a population-based screening study (China PEACE Million Persons Project). Lancet.

[23] Li J., Zhao F., Wang Y., Chen J., Tao J., Tian G., Wu S., Liu W., Cui Q., Geng B. (2017). Gut microbiota dysbiosis contributes to the development of hypertension. Microbiome.

[24] Yan Q., Gu Y., Li X., Yang W., Jia L., Chen C., Han X., Huang Y., Zhao L., Li P. (2017). Alterations of the Gut Microbiome in Hypertension. Front. Cell Infect. Microbiol..

[25] Huart J., Leenders J., Taminiau B., Descy J., Saint-Remy A., Daube G., Krzesinski J.M., Melin P., de Tullio P., Jouret F. (2019). Gut Microbiota and Fecal Levels of Short-Chain Fatty Acids Differ Upon 24-Hour Blood Pressure Levels in Men. Hypertension.

[26] Sun S., Lulla A., Sioda M., Winglee K., Wu M.C., Jacobs D.R., Shikany J.M., Lloyd-Jones D.M., Launer L.J., Fodor A.A. (2019). Gut Microbiota Composition and Blood Pressure. Hypertension.

[27] Santisteban M.M., Qi Y., Zubcevic J., Kim S., Yang T., Shenoy V., Cole-Jeffrey C.T., Lobaton G.O., Stewart D.C., Rubiano A. (2017). Hypertension-Linked Pathophysiological Alterations in the Gut. Circ. Res..

[28] Yang T., Santisteban M.M., Rodriguez V., Li E., Ahmari N., Carvajal J.M., Zadeh M., Gong M., Qi Y., Zubcevic J. (2015). Gut dysbiosis is linked to hypertension. Hypertension.

[29] Adnan S., Nelson J.W., Ajami N.J., Venna V.R., Petrosino J.F., Bryan R.M., Durgan D.J. (2017). Alterations in the gut microbiota can elicit hypertension in rats. Physiol. Genom..

[30] Okamoto K., Aoki K. (1963). Development of a strain of spontaneously hypertensive rats. Jpn. Circ. J..

[31] Enright E.F., Griffin B.T., Gahan C.G.M., Joyce S.A. (2018). Microbiome-mediated bile acid modification: Role in intestinal drug absorption and metabolism. Pharmacol. Res..

[32] Zhang J., Chen Y., Sun Y., Wang R., Zhang J., Jia Z. (2018). Plateau hypoxia attenuates the metabolic activity of intestinal flora to enhance the bioavailability of nifedipine. Drug Deliv..

[33] Berg J., Brandt K.K., Al-Soud W.A., Holm P.E., Hansen L.H., Sørensen S.J., Nybroe O. (2012). Selection for Cu-tolerant bacterial communities with altered composition, but unaltered richness, via long-term Cu exposure. Appl. Environ. Microbiol..

[34] Lan K., Su M., Xie G., Ferslew B.C., Brouwer K.L., Rajani C., Liu C., Jia W. (2016). Key Role for the 12-Hydroxy Group in the Negative Ion Fragmentation of Unconjugated C24 Bile Acids. Anal. Chem..

[35] Xie G., Wang X., Huang F., Zhao A., Chen W., Yan J., Zhang Y., Lei S., Ge K., Zheng X. (2016). Dysregulated hepatic bile acids collaboratively promote liver carcinogenesis. Int. J. Cancer.

[36] Ling Z., Shu N., Xu P., Wang F., Zhong Z., Sun B., Li F., Zhang M., Zhao K., Tang X. (2016). Involvement of pregnane X receptor in the impaired glucose utilization induced by atorvastatin in hepatocytes. Biochem. Pharmacol..

[37] Patki K.C., Von Moltke L.L., Greenblatt D.J. (2003). In vitro metabolism of midazolam, triazolam, nifedipine, and testosterone by human liver microsomes and recombinant cytochromes p450: Role of cyp3a4 and cyp3a5. Drug Metab. Dispos. Biol. Fate Chem..

[38] von Moltke L.L., Greenblatt D.J., Harmatz J.S., Shader R.I. (1993). Alprazolam metabolism in vitro: Studies of human, monkey, mouse, and rat liver microsomes. Pharmacology.

[39] Charni-Natan M., Goldstein I. (2020). Protocol for Primary Mouse Hepatocyte Isolation. STAR Protoc..

[40] Kamareddine L., Najjar H., Sohail M.U., Abdulkader H., Al-Asmakh M. (2020). The Microbiota and Gut-Related Disorders: Insights from Animal Models. Cells.

[41] Kyoung J., Atluri R.R., Yang T. (2022). Resistance to Antihypertensive Drugs: Is Gut Microbiota the Missing Link?. Hypertension.

[42] Yang X., Zhang X., Yang W., Yu H., He Q., Xu H., Li S., Shang Z., Gao X., Wang Y. (2021). Gut Microbiota in Adipose Tissue Dysfunction Induced Cardiovascular Disease: Role as a Metabolic Organ. Front. Endocrinol..

[43] Chen H.Q., Gong J.Y., Xing K., Liu M.Z., Ren H., Luo J.Q. (2021). Pharmacomicrobiomics: Exploiting the Drug-Microbiota Interactions in Antihypertensive Treatment. Front. Med..

[44] Doestzada M., Vila A.V., Zhernakova A., Koonen D.P.Y., Weersma R.K., Touw D.J., Kuipers F., Wijmenga C., Fu J. (2018). Pharmacomicrobiomics: A novel route towards personalized medicine?. Protein Cell.

[45] Flowers S.A., Bhat S., Lee J.C. (2020). Potential Implications of Gut Microbiota in Drug Pharmacokinetics and Bioavailability. Pharmacotherapy.

[46] Tuteja S., Ferguson J.F. (2019). Gut Microbiome and Response to Cardiovascular Drugs. Circ. Genom. Precis. Med..

[47] Choi M.S., Yu J.S., Yoo H.H., Kim D.H. (2018). The role of gut microbiota in the pharmacokinetics of antihypertensive drugs. Pharmacol. Res..

[48] Mishima E., Abe T. (2022). Role of the microbiota in hypertension and antihypertensive drug metabolism. Hypertens. Res. Off. J. Jpn. Soc. Hypertens..

[49] Mariat D., Firmesse O., Levenez F., Guimaraes V., Sokol H., Dore J., Corthier G., Furet J.P. (2009). The Firmicutes/Bacteroidetes ratio of the human microbiota changes with age. BMC Microbiol..

[50] Ley R.E., Turnbaugh P.J., Klein S., Gordon J.I. (2006). Microbial ecology: Human gut microbes associated with obesity. Nature.

[51] Kato R., Yuasa H., Inoue K., Iwao T., Tanaka K., Ooi K., Hayashi Y. (2007). Effect of Lactobacillus casei on the absorption of nifedipine from rat small intestine. Drug Metab. Pharmacokinet..

[52] Nassir F. (2022). NAFLD: Mechanisms, Treatments, and Biomarkers. Biomolecules.

[53] Toda T., Saito N., Ikarashi N., Ito K., Yamamoto M., Ishige A., Watanabe K., Sugiyama K. (2009). Intestinal flora induces the expression of Cyp3a in the mouse liver. Xenobiotica.

[54] Moore J.T., Kliewer S.A. (2000). Use of the nuclear receptor PXR to predict drug interactions. Toxicology.

[55] Xie W., Radominska-Pandya A., Shi Y., Simon C.M., Nelson M.C., Ong E.S., Waxman D.J., Evans R.M. (2001). An essential role for nuclear receptors SXR/PXR in detoxification of cholestatic bile acids. Proc. Natl. Acad. Sci. USA.

[56] Guengerich F.P., Martin M.V., Beaune P.H., Kremers P., Wolff T., Waxman D.J. (1986). Characterization of rat and human liver microsomal cytochrome P-450 forms involved in nifedipine oxidation, a prototype for genetic polymorphism in oxidative drug metabolism. J. Biol. Chem..

[57] Staudinger J.L., Goodwin B., Jones S.A., Hawkins-Brown D., MacKenzie K.I., LaTour A., Liu Y., Klaassen C.D., Brown K.K., Reinhard J. (2001). The nuclear receptor PXR is a lithocholic acid sensor that protects against liver toxicity. Proc. Natl. Acad. Sci. USA.

[58] Schuetz E.G., Strom S., Yasuda K., Lecureur V., Assem M., Brimer C., Lamba J., Kim R.B., Ramachandran V., Komoroski B.J. (2001). Disrupted bile acid homeostasis reveals an unexpected interaction among nuclear hormone receptors, transporters, and cytochrome P450. J. Biol. Chem..

[59] Watkins R.E., Wisely G.B., Moore L.B., Collins J.L., Lambert M.H., Williams S.P., Willson T.M., Kliewer S.A., Redinbo M.R. (2001). The human nuclear xenobiotic receptor PXR: Structural determinants of directed promiscuity. Science.

[60] LeCluyse E.L. (2001). Pregnane X receptor: Molecular basis for species differences in CYP3A induction by xenobiotics. Chem.-Biol. Interact..

[61] Mun S.J., Lee J., Chung K.S., Son M.Y., Son M.J. (2021). Effect of Microbial Short-Chain Fatty Acids on CYP3A4-Mediated Metabolic Activation of Human Pluripotent Stem Cell-Derived Liver Organoids. Cells.

[62] Bhutia Y.D., Ogura J., Sivaprakasam S., Ganapathy V. (2017). Gut Microbiome and Colon Cancer: Role of Bacterial Metabolites and Their Molecular Targets in the Host. Curr. Color. Cancer Rep..

[63] Liu J., Cheng Y., Zhang Y., Huang S., Liu Z., Wang X. (2021). Lactobacillus rhamnosus induces CYP3A and changes the pharmacokinetics of verapamil in rats. Toxicol. Lett..

[64] Yang Z., Wang Q., Liu Y., Wang L., Ge Z., Li Z., Feng S., Wu C. (2023). Gut microbiota and hypertension: Association, mechanisms and treatment. Clin. Exp. Hypertens..

