# Supplementary material for: Effect of Gut Microbiota on the Pharmacokinetics of Nifedipine in Spontaneously Hypertensive Rats
Source: Pharmaceutics. 2023 Aug 3;15(8):2085. doi: 10.3390/pharmaceutics15082085 (PMC10458652; doi:10.3390/pharmaceutics15082085)
Supplement: Supplementary file 1 [file pharmaceutics-15-02085-s001.zip › pharmaceutics-2500704-supplementary.pdf]

Supplementary Material

Supplementary Figures and Tables

Supplementary Figure

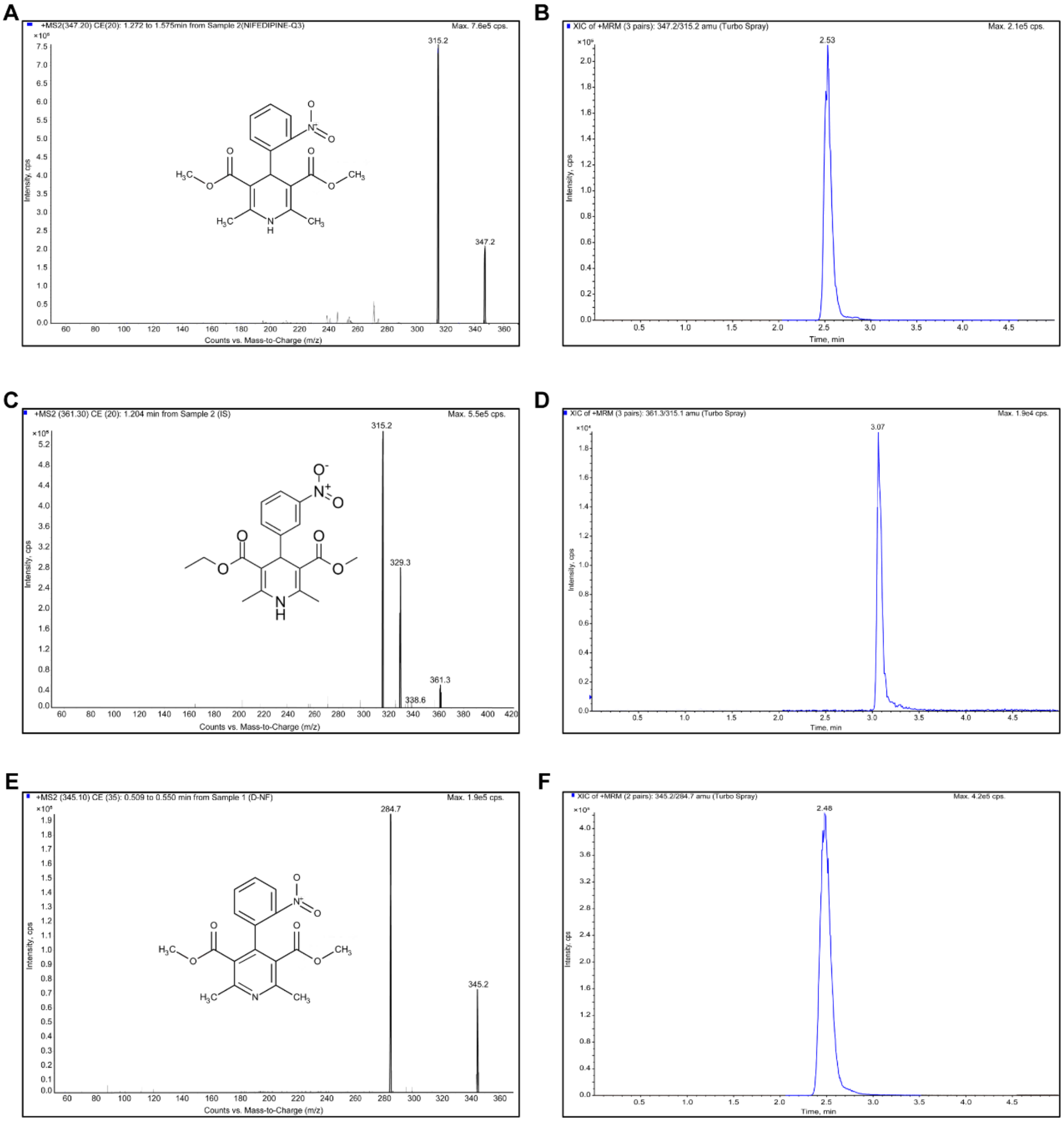

Supplementary Figure S1. Product ion spectrum of (A) nifedipine, (C) nitrendipine and (E) dehydronifedipine; HPLC chromatographs of (B) nifedipine, (D) nitrendipine and (F) dehydronifedipine.

Supplementary Table

Supplementary Table S1. Primer sequences used for RT-PCR.

Gene	Forward primer (5′→3′)	Reverse primer (5′→3′)
rGAPDH	TGAACGGGAAGCTCACTGG	GCTTCACCACCTTCTTGATGTC
rCYP3A1	GATGTTGAAATCAATGGTGTG	TTCAGAGGTATCTGTGTTTCC
rPXR	CTTGGAGGGAGGTTGGTAGTT	CTGCCACATGGAGTCAAATCA
rMdr1a	AGGCAATGGCAACATTTTTTGGTGG	GATAAGCAGAAAAGCTGCACCCATG
